# Supplementary material for: The genome sequence of the oak pinhole borer, Platypus cylindrus (Fabricius, 1792)
Source: Wellcome Open Res. 2024 Oct 18;9:305. Originally published 2024 Jun 11. [Version 2] doi: 10.12688/wellcomeopenres.22425.2 (PMC11411242; doi:10.12688/wellcomeopenres.22425.2)
Supplement: Peer review history

# Reviewer report: Reviewer response for version 2

**DOI:** 10.21956/wellcomeopenres.25596.r114831

**Published:** 2024-12-26

**Authors:** Badr M. Al-Shomrani

**Affiliations:** 1 King Abdulaziz City for Science and Technology, Riyadh, Saudi Arabia

The study provides a chromosomal-level genome sequence for a male *Platypus cylindrus* with a high BUSCO completeness score of 96.2%. This work is a valuable addition**** to the Darwin Tree of Life Project. The authors employed a combination of PacBio HiFi sequencing and Hi-C data to improve assembly quality and accuracy, marking a notable advancement in the genomic understanding of *Platypus cylindrus*.

Are sufficient details of methods and materials provided to allow replication by others?

Yes

Is the rationale for creating the dataset(s) clearly described?

Yes

Are the datasets clearly presented in a useable and accessible format?

Yes

Are the protocols appropriate and is the work technically sound?

Yes

Reviewer Expertise:

Bioinformatics

I confirm that I have read this submission and believe that I have an appropriate level of expertise to confirm that it is of an acceptable scientific standard.

# Reviewer report: Reviewer response for version 2

**DOI:** 10.21956/wellcomeopenres.25596.r110573

**Published:** 2024-12-12

**Authors:** Nathan Rank

**Affiliations:** 1 Sonoma State University, Rohnert Park, California, USA

The paper is well written. I missed some mention of the fact that these beetles have some interesting ecological relationships with fungi that are not described well in the introduction. The introduction also mentions that their distribution is restricted to Eurasia, and lists a collection of European countries, but the genus is also found in California and North America. A casual reader might think that the distribution is limited to Eurasia. The ecological relationships that I find interesting are the interactions with fungi, which are referenced in many papers mentioning the genus. Additionally, bark beetles involved in vectoring tree diseases such as Sudden Oak Death belong to the same subfamily as the ones whose genome is presented here. 

 It would be nice if an approximate body size scale were included along with the image of adult beetles; they are smaller than one might expect but you can't tell that from the figure. One other item that I would mention in review is that the methods used to reconstruct the mitochondrial sequence can sometimes create errors in the sequence itself or the annotation. I had to make a lot of manual adjustments to mitochondrial sequences automatically, and if I hadn't done that, there would have been mitochondrial proteins without stop codons and the ribosomal RNA genes would have been incompletely listed. Hopefully the authors also compared the mitochondrial genome they generated with some close relatives where the genome was carefully made and annotated, otherwise I believe errors could be present.

Are sufficient details of methods and materials provided to allow replication by others?

No

Is the rationale for creating the dataset(s) clearly described?

Yes

Are the datasets clearly presented in a useable and accessible format?

Yes

Are the protocols appropriate and is the work technically sound?

Partly

Reviewer Expertise:

Evolutionary ecology, genetics, mitonuclear interactions. I am not so competent in the genome assembly methods, except for mitochondrial genes as mentioned above.

I confirm that I have read this submission and believe that I have an appropriate level of expertise to confirm that it is of an acceptable scientific standard, however I have significant reservations, as outlined above.

# Reviewer report: Reviewer response for version 2

**DOI:** 10.21956/wellcomeopenres.25596.r106132

**Published:** 2024-11-26

**Authors:** Sophie Tandonnet

**Affiliations:** 1 Universitat de Barcelona, Barcelona, Catalonia, Spain

The amendments seem incomplete as only the first suggestion I made was added to the article.

 Not all points need to be addressed but it would be useful to add more information of how the sex chromosomes were identified (and, related to this, how /if the Y chromosome was concatenated into a single scaffold, which seems the case in the assembly file). Additionally, the transcriptomic data mentioned in the PRJEB59383 project doesn't seem available. But maybe this will be added in the future.

Are sufficient details of methods and materials provided to allow replication by others?

Partly

Is the rationale for creating the dataset(s) clearly described?

Yes

Are the datasets clearly presented in a useable and accessible format?

Yes

Are the protocols appropriate and is the work technically sound?

Yes

Reviewer Expertise:

Evolutionary Biology, Genetics, sex determination, bioinformatics, non-model organisms

I confirm that I have read this submission and believe that I have an appropriate level of expertise to confirm that it is of an acceptable scientific standard.

# Reviewer report: Reviewer response for version 1

**DOI:** 10.21956/wellcomeopenres.24704.r95872

**Published:** 2024-09-18

**Authors:** Luciano Palmieri

**Affiliations:** 1 Competence Centre for Plant Health, Free University of Bozen-Bolzano, Bolzano, BZ, Italy

This manuscript presents the genome assembly of *Platypus cylindrus*, as part of the Darwin Tree of Life Project, which aims to sequence all named eukaryotic species in Britain and Ireland. The assembled genome spans 147.5 Mbases, with most of the sequence scaffolded into 8 chromosomal pseudomolecules, including the X and Y sex chromosomes. Additionally, the mitochondrial genome (19.29 Kbases) was successfully assembled, and automatic gene annotation identified 13,468 protein-coding genes. The genome was sequenced using PacBio HiFi long reads from a male specimen, and scaffolded with Hi-C data. The assembly is of high quality, with a BUSCO completeness score of 96.2%.

 This study is part of a growing body of work from the same group, which has developed and refined an efficient pipeline for generating high-quality genome assemblies. This pipeline has been used to describe the genomes of dozens of insect species, making significant contributions to our understanding of insect  genomics. The rationale for creating this dataset is not often clear, while it provides valuable data for understanding the genetics of *P. cylindrus*, the broader ecological and evolutionary context of this species is less emphasized. Nonetheless, the described genome provides a foundational resource for further research into the genetics, evolution, and ecological role of this species.

Are sufficient details of methods and materials provided to allow replication by others?

Yes

Is the rationale for creating the dataset(s) clearly described?

Partly

Are the datasets clearly presented in a useable and accessible format?

Yes

Are the protocols appropriate and is the work technically sound?

Yes

Reviewer Expertise:

ColeopteraSystematicsBiogeographyBiodiversityTaxonomyEntomologySystematic EntomologyInsect TaxonomyPhylogenetic AnalysisEvolutionInsect IdentificationNatural HistoryScientific Digital PhotographySpecies DiversityPhylogeneticsMolecular PhylogeneticsMolecular TaxonomyMolecular SystematicsInvertebrate ZoologyZoological NomenclatureFicusParasitoidsMacroevolutionGenome AssemblyPhylogenomics

I confirm that I have read this submission and believe that I have an appropriate level of expertise to confirm that it is of an acceptable scientific standard.

# Reviewer report: Reviewer response for version 1

**DOI:** 10.21956/wellcomeopenres.24704.r95869

**Published:** 2024-09-04

**Authors:** Sophie Tandonnet

**Affiliations:** 1 Universitat de Barcelona, Barcelona, Catalonia, Spain

In this note, the authors present a highly complete and continuous genome assembly for the species *Platypus cylindrus.* It is an addition to the growing database of high quality genomes coming from the Darwin Tree of Life project. The note is well written and the data correctly referenced and available. The annotation is available containing 13,468 protein coding genes.

**Below are some suggestions, questions and general comments**

- minor comment: I missed reading more background on this species: ecology, special characteristics, lifestyle, habitat, etc…

- The abstract states that "Most of the assembly is scaffolded into 8 chromosomal pseudomolecules, including the X and Y sex chromosomes." but later in the report it is said "Y chromosome scaffolds were identified but were not scaffolded". Looking at the PRJEB60808 project page on ENA, I did find the Y chromosome as a single sequence. So I'm confused: is the Y chromosome on ENA only a part of the Y? Or were all the scaffolds tagged as Y collated together?  Could the authors clarify this point?
- Related to the previous point: how were the X and Y chromosome material identified? Was a coverage analysis performed? Was it based on synteny? If so, with which species? I think it is very cool to have the Y chromosome (which is usually hard to assemble well) and it may be useful for other researchers to have more information on how the Y was identified.

- Add "from an additional female specimen" in the sentence "Primary assembly contigs were scaffolded with chromosome conformation Hi-C data." in the report section. This is because other assemblies have both the HiC and the Pacbio/10X sequencing from the same individual.
- For the genome annotation: please add the protein database or transcriptome data used as this can influence the completeness of the annotation. Is 13,468 protein coding genes around the expected number of genes for a beetle? What is the proteome BUSCO score?
- The PRJEB59383 page states that "This project collects the genomic and transcriptomic data generated for Platypus cylindrus". However I couldn't locate the transcriptomic data. 
- The MT genome is 19.29 kilobases in length: did the authors check for internal duplication in this sequence?
- Figure 5: Why are there "white bands" (no contacts) in the Hi-C contact map?
- Sample acquisition and nucleic acid extraction: the text seems to relate to the handling of the male sample. How was the female sample handled for HiC data generation?

Are sufficient details of methods and materials provided to allow replication by others?

Partly

Is the rationale for creating the dataset(s) clearly described?

Yes

Are the datasets clearly presented in a useable and accessible format?

Yes

Are the protocols appropriate and is the work technically sound?

Yes

Reviewer Expertise:

Evolutionary Biology, Genetics, sex determination, bioinformatics, non-model organisms

I confirm that I have read this submission and believe that I have an appropriate level of expertise to confirm that it is of an acceptable scientific standard, however I have significant reservations, as outlined above.

# Reviewer report: Reviewer response for version 1

**DOI:** 10.21956/wellcomeopenres.24704.r89813

**Published:** 2024-07-26

**Authors:** Robert Angus

**Affiliations:** 1 Department of Life Sciences (Insects), The Natural History Museum, London, UK

These authors present this piece of work as part of the Natural History Museum’s project to accumulate DNA genotypes of all British animals. As such it is a valuable contribution, based on a recently captured reliably identified beetle. It is a straightforward account using their standard protocols.

 A sound and useful piece of work.

Are sufficient details of methods and materials provided to allow replication by others?

Yes

Is the rationale for creating the dataset(s) clearly described?

Yes

Are the datasets clearly presented in a useable and accessible format?

Yes

Are the protocols appropriate and is the work technically sound?

Yes

Reviewer Expertise:

NA

I confirm that I have read this submission and believe that I have an appropriate level of expertise to confirm that it is of an acceptable scientific standard.
